# Therapeutic Effect of Bone Marrow Mesenchymal Stem Cells on Laser-Induced Retinal Injury in Mice

**DOI:** 10.3390/ijms15069372

**Published:** 2014-05-27

**Authors:** Yuanfeng Jiang, Yan Zhang, Lingjun Zhang, Meiyan Wang, Xiaomin Zhang, Xiaorong Li

**Affiliations:** 1 Tianjin Medical University Eye Hospital and Eye Institute, No. 251, Fukang Road, Nankai District, Tianjin 300384, China; E-Mails: frankjyf@126.com (Y.J.); yanzhang9927@163.com (Y.Z.); zhanglingjun08@163.com (L.Z.); 2 Department of Ophthalmology, Haibin People’s Hospital of Tianjin, No. 400, Chuangye Road, Binhai New District, Tianjin 300280, China; E-Mail: tongxin1506@sina.com

**Keywords:** mesenchymal stem cells, retina, apoptosis, glial fibrillary acidic protein (GFAP), matrix metalloproteinase-2 (MMP-2)

## Abstract

Stem cell therapy has shown encouraging results for neurodegenerative diseases. The retina provides a convenient locus to investigate stem cell functions and distribution in the nervous system. In the current study, we investigated the therapeutic potential of bone marrow mesenchymal stem cells (MSCs) by systemic transplantation in a laser-induced retinal injury model. MSCs from C57BL/6 mice labeled with green fluorescent protein (GFP) were injected via the tail vein into mice after laser photocoagulation. We found that the average diameters of laser spots and retinal cell apoptosis were decreased in the MSC-treated group. Interestingly, GFP-MSCs did not migrate to the injured retina. Further examination revealed that the mRNA expression levels of glial fibrillary acidic protein and matrix metalloproteinase-2 were lower in the injured eyes after MSC transplantation. Our results suggest that intravenously injected MSCs have the ability to inhibit retinal cell apoptosis, reduce the inflammatory response and limit the spreading of damage in the laser-injured retina of mice. Systemic MSC therapy might play a role in neuroprotection, mainly by regulation of the intraocular microenvironment.

## 1. Introduction

Many inherited retinal and retinal-neuronal degenerative diseases, such as retinitis pigmentosa, age-related macular disease (AMD), glaucoma and related retinal dystrophies, lead to retinal cell loss [1,2,3,4]. The death of retinal cells, especially the photoreceptors, cannot be reversed, because of the non-renewable character of neurocytes. Furthermore, no effective therapies have been developed to prevent or reverse the degenerative processes of these disorders.

Stem cell therapy has emerged as a novel and promising candidate approach for the treatment of neurological disease, probably by endogenous neurogenesis, decreasing apoptosis, cell replacement and modulation on inflammation and immune response [5,6]. Mesenchymal stem cells (MSCs), progenitors of all connective tissue cells, are an attractive stem cell source for the treatment of neurological disease. Recently, MSC transplantation has demonstrated significant neuroprotection in several degenerative models of the central nervous system [7,8,9]. For example, intravitreal MSC transplantation increases the survival of retinal ganglion cell axons in experimental glaucoma [10]. MSCs secrete factor(s) that promote photoreceptor cell survival in Royal College of Surgeon (RCS) rats [11]. Moreover, systemically administered MSCs might play an important role in the wound modulation of physically damaged retinal tissues [12]. Importantly, MSCs provide an autologous approach for cell-based therapies.

In the current study, we used a mouse model of laser-induced retinal injury to investigate the therapeutic potential of MSCs for the treatment of these diseases. After retinal injury, green fluorescent protein (GFP)-labeled MSCs were transplanted via the tail vein, following by examination of morphological changes, retina cell apoptosis and the mRNA expression of glial fibrillary acidic protein (GFAP) and matrix metalloproteinase (MMP)-2.

## 2. Results

### 2.1. Characterization of Green Fluorescent Protein Labeled Marrow Mesenchymal Stem Cells

**Figure 1:**
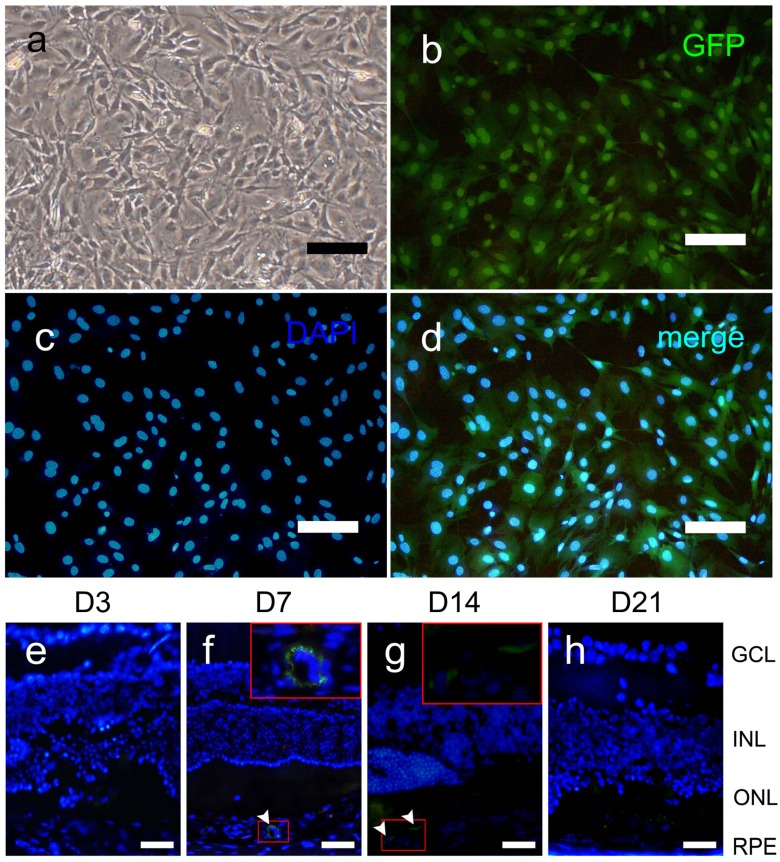
Green fluorescent protein (GFP) expression of cultivated GFP-labeled mesenchymal stem cells (MSCs) and their trace in the retina. (**a**) Fibroblastic phenotype of Passage 15 MSCs under an inverted phase contrast microscope; (**b**) Green fluorescence of GFP in the cytoplasm under a fluorescence microscope; (**c**) Blue fluorescence of 4',6-diamidino-2-phenylindole (DAPI) in the nucleus; (**d**) Merged image of (**b**) and (**c**); Frozen sections of the eyes were observed under a fluorescence microscope at each time point (**e**–**h**); Green GFP+ MSCs are indicated by white arrowheads (**f**,**g**). Scale bars: 50 μm.

Cells in culture formed an adherent monolayer on plastic flasks and displayed the traits of MSCs, including a typical fibroblast-like morphology and strong proliferative ability, even after 15 passages (Figure 1a). More than 95% of the cells expressed GFP in their cytoplasm and exhibited green fluorescence in the excitation wavelength of 493 nm (Figure 1b–d).

### 2.2. GFP-MSC Migration

To determine whether MSCs migrate to the eye and remain there after systemic injection, we examined GFP+ MSCs in the eyes of the MSC-treated group for up to 21 days. GFP+ cells were not detected in the retinas at any time point (Figure 1e–h). Only very few GFP+ cells were found in subretinal areas or inside the choroid capillaries in two mice both on Days 7 and 14 (Figure 1f,g). Additionally, the GFP+ cells were only observed in one or two laser spots among 15–20 laser spots in each eye.

**Figure 2:**
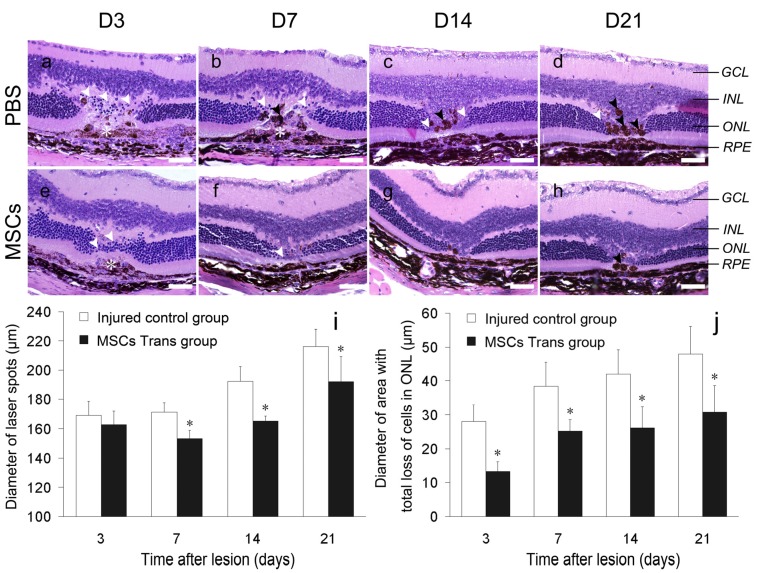
Histopathological changes of laser-injured retina (hematoxylin and eosin staining) and morphometric parameters of laser spots. (**a**,**e**) At three days after injury, the retinal pigment epithelial (RPE) layer was severely disrupted, and extensive pyknosis (white arrowheads) was seen in the outer nuclear layer (ONL). The acellular membrane (asterisk) included pigment cells and inflammatory cells in both groups at Day 3 (**a**,**e**), which still existed in the injured control group and disappeared in the MSC-treated group at Day 7 (**b**,**f**). MSC-treated retinas showed less proliferative gliocytes, pigmentation and scar tissue (black arrowheads) (**g**,**h**) than that in the injured control group (**c**,**d**) at Days 14 and 21. Values are expressed as the means ± SD (**i**,**j**). Asterisks denote significant differences between values obtained at the same time point in the two groups (*, *p* < 0.05). Scale bars: 50 µm. GCL, ganglion cell layer; INL, inner nuclear layer.

### 2.3. Histopathological Changes in the Retina after Laser Injury

Light microscopy revealed the typical histopathological development of argon laser-induced retinal lesions [13] in the injured control group (Figure 2a–d). However, improvement of the retinal tissue structure was found in the MSC-treated group (Figure 2e–h). At three days after laser photocoagulation, mild edema of the outer nuclear layer (ONL) of the retina and loss of pigment in the retinal pigment epithelial (RPE) layer were observed at the laser spots. The ONL was disturbed, especially at the center of the lesion, because of the loss and massive pyknosis of nuclei. The structure of the inner and outer segments of photoreceptors was disrupted and replaced by a cellular membrane consisting of proliferating RPE cells, pigment-laden cells and dispersed pigment granules (Figure 2a). Mice treated with MSCs showed greatly reduced retinal disorganization caused by laser injury with more residual photoreceptor cells and less disrupted inner and outer segments than those in mice without MSC treatment (Figure 2e).

The extent of laser spots and the loss of cells in the ONL were gradually increased over time. After Days 7, 14 and 21, the defective areas in the injured control group were replaced by massive gliocytes and RPE cells, which were hyperplastic and hypertrophic (Figure 2b–d). In contrast, MSC treatment reduced the range of the subretinal membrane in the injured spot and preserved the retinal tissue structure with less scar tissue, pigmentation, hyperplasia and hypertrophy (Figure 2f–h).

At three days after laser photocoagulation, statistical analysis showed that the average diameter of areas with a total loss of cells in the ONL of the MSC-treated group was smaller than that in the injured control group (*t* = 6.4909, *p* < 0.05), while the average diameter of laser spots showed no significant difference (*t* = 1.13, *p* > 0.05). At seven, 14 and 21 days after laser injury, both the average diameters of laser spots (*t* = 5.077, 6.486, 2.845) and areas with a total loss of cells in the ONL (*t* = 4.088, 4.046, 3.693) were reduced in the MSC-treated group compared with those in the control group (*p* < 0.05) (Figure 2i,j).

### 2.4. Apoptosis of Retinal Cells

**Figure 3:**
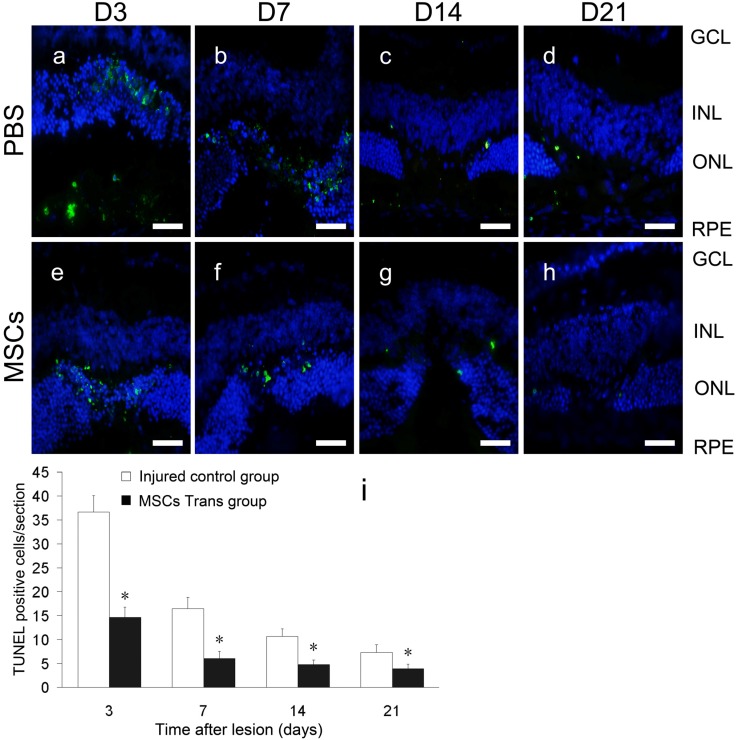
Apoptosis of retinal cells at laser spots in mice treated with or without MSCs. Retinas of the injured control group (**a**–**d**) and MSC-treated group (**e**–**h**). Green indicates terminal deoxynucleotidyl transferase-mediated dUTP-biotin nick-end labeling (TUNEL)+ cells. Blue indicates DAPI-stained nuclei. Scale bars: 50 μm. The average numbers of apoptotic cells were calculated and found to be significantly reduced in the MSC-treated group at all time points compared with those in the injured control group (**i**). *, *p* < 0.05. Scale bars: 50 µm. GCL, ganglion cell layer; INL, inner nuclear layer.

After laser photocoagulation, terminal deoxynucleotidyl transferase-mediated dUTP-biotin nick-end labeling (TUNEL)+ cells were examined in the lesions for up to 21 days by green fluorescence of the apoptotic nuclei under a fluorescence microscope (Figure 3a–h). On Day 3, a large number of TUNEL+ cells were observed in the control group, mainly in the ONL and RPE layers. On Days 7, 14 and 21, most TUNEL+ cells were located at the edge of the lesions in the ONL. In the INL and GCL, there were sporadic TUNEL+ cells that could be detected. However, the average numbers of TUNEL+ cells were significantly reduced in the MSC-treated group at all time points (Figure 3i) (*t* = 13.293, 9.084, 8.011 and 4.567, *p* < 0.05). As expected, no apoptosis was observed in the retina of the negative control or normal control groups (data not shown).

### 2.5. GFAP and MMP-2 mRNA Expression

**Figure 4:**
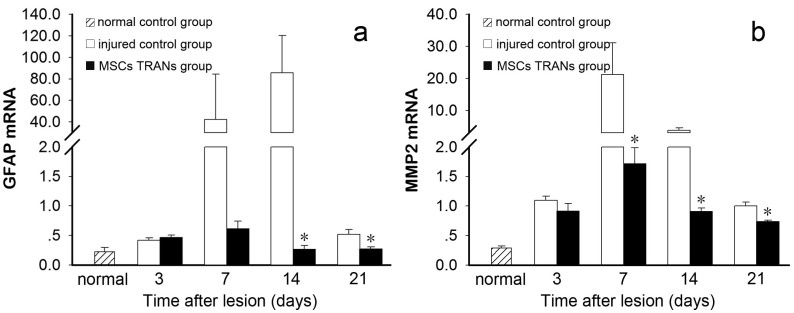
mRNA expression of GFAP and MMP-2 in laser-injured eyes. A parabolic change in GFAP (**a**) and MMP-2 (**b**) mRNA expression was found over time after laser injury. Asterisks denote significant differences between the injured control and MSC-treated groups (*, *p* < 0.05).

To determine whether systemic injection of MSCs changed the intraocular microenvironment, we examined the gene expression of GFAP and MMP-2, which are related to retinal inflammation and apoptosis. The results of real-time quantitative RT-PCR showed that the mRNA expression level of GFAP was increased in the retina after laser photocoagulation. The mRNA expression level of GFAP was also markedly increased at Day 7, peaked at Day 14 and then decreased at Day 21, but was still higher than the normal level. In contrast, the mRNA expression level of GFAP was not increased at Days 7 or 14 in the MSC-treated group (Figure 4a). In addition, the mRNA expression level of MMP-2 had started to rise at Day 3 after laser photocoagulation and then peaked at Day 7, whereas the mRNA expression level of MMP-2 in the MSC-treated group was significantly reduced at Days 7, 14 and 21 (Figure 4b).

## 3. Discussion

The laser injury model of the retina has been previously used by many researchers, and the mechanism and pathophysiology have been well studied for laser-induced damage [13,14,15,16,17]. Because MSCs migrate to injury sites following intravitreous injection, which results in retinal repair [18,19,20], we tested whether MSCs exhibit the same recruitment and protection upon laser injury when transplanted into mice intravenously. Our results showed that the retinal morphological structure was improved and retinal cell apoptosis was reduced after intravenous MSC transplantation. However, MSCs did not migrate into the retina by this administration route in our model.

Loss of photoreceptor cells, characterized by apoptosis, is the endpoint of most retinal degenerative diseases and the major cause of a decrease or even the loss of visual function in patients. Numerous strategies have been attempted to stop or repair the degeneration or loss of photoreceptors, including nutritional support therapy, gene therapy, the application of nerve growth factor and the development of an artificial eye [21,22,23,24,25]. These methods provide protection and restoration to some extent, but they cannot achieve satisfactory results, because of their limitations in clinical application and poor efficacy.

Stem cell transplantation has been recently applied to treat retinal diseases and has achieved initial results [26]. The multipotency of MSCs and their easy accessibility, high expansion potential and immune-privileged properties make them an ideal source for autologous transplantation aimed at cell-based therapy for clinical application [11,27]. It is assumed that the transplanted cells improve retinal impairment and cellular deficiency, because they provide neuroprotection, wound healing, anti-inflammatory effects and even cell replacement [11,28,29]. In RCS rats, culture medium conditioned by MSCs delays photoreceptor cell apoptosis, as demonstrated *in vivo* by histological and electrophysiological analyses after subretinal injection [11]. MSC transplantation in animal models of glaucoma shows neuroprotection by reducing ganglion cell apoptosis [29,30].

In our study, the laser-induced retinal injury was histologically improved by intravenous MSC transplantation. At the early stage, there was a reduction of inflammatory response revealed by less disorganization of retinal structure, milder edema and more residual nuclei in the ONL. In the later chronic phase, the spread of damage was limited, with less pigment deposition and scar tissue. More importantly, the apoptosis of retinal cells, especially photoreceptors, was significantly reduced throughout the course of MSC therapy. Hence, MSC therapy may relieve the acute inflammatory response and limit the expansion of scotoma after retinal photocoagulation treatment in neovascularization diseases, such as AMD and diabetic retinopathy [31,32].

We next determined whether the improvements observed in this model were caused by cell replacement. The results in Figure 5 showed that GFP-MSCs were not detected in the retina, suggesting that MSCs did not migrate to the injured retina. However, in several similar studies, MSCs migrated into the retina and expressed markers of RPE cells, endothelial cells, pericytes and photoreceptor cells [18,19]. MSCs were found mainly in the vitreous cavity after intravitreal transplantation in a rat glaucoma model, although a small proportion of discrete cells migrated into the host retina and was neuroprotective [10]. Subretinal transplantation into the RCS rat also showed improvement of retinal degeneration and preservation of retinal function [11]. However, Chung and colleagues have reported that systemically administered GFP-labeled MSCs were observed at the transitional zone between the damaged and normal retina at five weeks after retinotomy applied by a neodymium-doped yttrium aluminum garnet (Nd:YAG) laser [12]. Because the laser energy we used in this study did not break Brunch’s membrane of the retina, we believe that the integrity of the blood-retinal barrier may confine GFP-MSC migration to the injury and inflammatory sites when MSCs are injected systemically. We speculate that MSCs play a role in neuroprotection and damage repair by mainly regulating the intraocular microenvironment.

Our study further showed that MSC treatment reduced the mRNA expression of GFAP and MMP-2. GFAP is an intermediate filament protein expressed by numerous cell types of the central nervous system. The expression of GFAP in Müller cells of the retina is closely related to the severity and extent of retinal damage. Early studies in different species showed that laser injury increases GFAP expression and gliocyte proliferation, which were confirmed in our study by histological observation and quantitative RT-PCR [33]. In the later phase of laser injury, the decreased level of GFAP and limited hyperproliferative activity of gliocytes after MSC treatment further showed that MSCs might reduce or delay the formation of scar tissue and provide a stable microenvironment that is conducive for reconstruction of the injured retina. MMP-2, also known as gelatinase A, is involved in the breakdown of the extracellular matrix (ECM) in normal physiological and many disease processes, including wound healing, tissue remodeling, inflammatory responses, apoptosis, as well as cell differentiation and proliferation [34]. MMP-2 is rarely detected in the normal eye [35,36,37]. However, under pathological conditions, the expression level of MMP-2 is raised by stimulating factors, such as tissue injury and inflammation [38,39]. Disruption of the ECM caused by an imbalance of MMP-2 and its inhibitors leads to a further series of histopathological changes, including inflammatory infiltration and retinal cell apoptosis [40,41,42]. Previous studies of experimental autoimmune uveitis have demonstrated that MSCs display their immunomodulatory and therapeutic properties in a paracrine fashion [43,44]. The reduced expression of MMP-2 mRNA in the MSC-treated group was also probably attributed to the paracrine effects of MSCs by secreting neuroprotective factors and/or factors that can downregulate inflammation. It is also possible that MSCs directly regulate the expression of MMP-2. These results suggest that MSCs might play a role in preserving the homeostasis of the ECM, which is beneficial for the integrity of the retina and tissue repair. Further studies are needed to explore the specific mechanisms of MSCs in retinal repair.

## 4. Experimental Section

### 4.1. Animals

A total of 153 female C57BL/6 (B6) mice at 6–8 weeks of age were purchased from Peking University Health Science Center. All of the experimental procedures were approved by Institutional Animal Care and Use Committee (IACUC) of Tianjin Medical University (Permit Number: SYXK 2009-0001), and were in accord with the Association For Research In Vision And Ophthalmology Statement for the Use of Animals in Ophthalmic and Vision Research. Animals used in this study were maintained in a 12-h light/dark cycle and fed a chow diet with free access to drinking water in the animal facilities of Tianjin Medical University Eye Institute.

### 4.2. Study Design

B6 mice were randomly divided into three groups, including the injured control group (*n* = 68), MSC-treated group (*n* = 68) and normal control group (*n* = 17). At 1 day after photocoagulation, 1 × 10^6^ MSCs in 200 μL PBS were intravenously injected via the tail vein into each mouse of the MSC-treated group. Mice in the injured control group were administered an equal volume of PBS. Animals were sacrificed at 3, 7, 14 and 21 days after laser injury. The procedures were carried out under anesthesia.

### 4.3. Laser-Induced Retinal Injury Model

Mice were anesthetized by an intraperitoneal injection of chloral hydrate (500 mg/kg). Their pupils were dilated with 0.5% tropicamide and 0.5% phenylephrine hydrochloride. A glass slide was used to flatten the cornea to obtain a clear view of the fundus. With the aid of a multi-wavelength laser slit lamp (NOVUS OMNI, Coherent, Santa Clara, CA, USA), 15–20 argon laser spots (150 mw, 100 ms, 100 μm) were applied to each fundus at about two disc diameters away from the optic disc, while avoiding the major vessels.

### 4.4. GFP Labeled MSCs

GFP-labeled MSCs derived from the bone marrow of B6 mice at Passage 6 were purchased from Cyagen Biosciences (Guangzhou, China). The cells were seeded on 25-cm^2^ tissue culture flasks in growth medium (Cyagen Biosciences) and incubated at 37 °C in a humidified atmosphere with 5% CO_2_. The growth medium was changed every other day. Passages 8–10 MSCs were adjusted to 5 × 10^6^ cells/mL for transplantation.

### 4.5. Evaluation of GFP Expression in MSCs

MSCs were seeded on 10 × 10 mm coverslips, and 1 μg/mL 4',6-diamidino-2-phenylindole (DAPI; Sigma, St. Louis, MO, USA) was applied to the cells at confluence. GFP expression was observed under a fluorescence microscope (DP73; OLYMPUS, Tokyo, Japan) at an excitation wavelength of 493 nm.

### 4.6. Tracing GFP-Labeled MSCs

At 3, 7, 14 and 21 days after laser injury, frozen sections of the eyes from the MSC-treated group were prepared and stained with DAPI. The location of GFP-labeled MSCs in the eye was examined under a fluorescence microscope.

### 4.7. Histopathological Analysis of the Laser-Injured Retina

To examine morphological changes, serial sections (3 μm) were stained with hematoxylin and eosin and observed under a light microscope. The diameters of laser spots were determined by measuring the widest area of retinal tissue disruption. The diameters of laser spots and areas with a total loss of cells in the ONL were analyzed by image processing software (CellSens Standard 1.6; Olympus, Tokyo, Japan) in a blinded manner. Sections with the maximum diameter of each laser spot were selected for subsequent analysis. Six views of laser spots were randomly chosen in each eye (*n* = 6 per group) and used to estimate the average value.

### 4.8. Apoptosis Analysis of the Laser-Injured Retina

TUNEL were performed on frozen sections (8 μm) of the eye to examine cellular apoptosis according to the manufacturer’s protocol (*In Situ* Cell Death Detection Kit, Fluorescein; Roche Applied Science, Mannheim, Germany). The specificity of the TUNEL assay was tested by staining the sections with the labeling solution without terminal transferase (negative control). In addition, a positive control was prepared by treating the sections with DNase I (Thermo Scientific, Tewksbury, MA, USA). All sections were counterstained with DAPI after the TUNEL reaction. Finally, the sections were mounted with antifade mounting medium and analyzed under the fluorescence microscope. Two images of the same spot were captured and matched to identify the TUNEL-positive nuclei. Four views of laser spots were randomly chosen in each eye (*n* = 6 per group) and used to estimate the average value.

### 4.9. Real-Time Quantitative RT-PCR

Total RNA was extracted from the eyes of each mouse (*n* = 5 per group) using a GeneJET RNA Purification kit (Thermo) according to the manufacturer’s instructions. cDNA was synthesized by a reverse transcription kit (RevertAid First Strand cDNA Synthesis Kit; Thermo), according to the manufacturer’s instructions. To detect the mRNA expression of GFAP and MMP-2, real-time quantitative RT-PCR was performed with SYBR Green (Thermo) and the 7900HT Fast Real-Time PCR system (Life Technologies, Gaithersburg, MD, USA). Glyceraldehyde 3-phosphate dehydrogenase (GAPDH) was used as the internal control. Primer sequences for mouse transcripts are shown in Table 1. PCR conditions were 2 min at 50 °C, 10 min at 95 °C, followed by 40 cycles of 15 s at 95 °C and 1 min at 60 °C. Melting curve analysis to determine the dissociation of PCR products was performed between 60 and 95 °C. Four points of a 10-fold serial dilution of standard DNA were used for absolute quantification. Data were analyzed with the following formula: standardization of target gene expression = the amount of target gene expression (GFAP or MMP-2)/the amount of reference gene expression (GAPDH).

**Table 1:** Primers used for quantitative polymerase chain reaction.

Gene Name	Forward Primer	Reverse Primer
*GFAP*	TGGAGGTGGAGAGGGACAAC	TGGTTTCATCTTGGAGCTTCTG
*MMP-2*	GAGGACTATGACCGGGATAAGAAGT	GGGCACCTTCTGAATTTCCA
*GAPDH*	TGTGTCCGTCGTGGATCTGA	CCTGCTTCACCACCTTCTTGA

### 4.10. Statistical Analysis

All values are expressed as means ± SD. Data were analyzed using the Student’s *t*-test. A *p*-value of less than 0.05 was considered to be statistically significant.

## 5. Conclusions

Similar to local injection of MSCs, our study demonstrated that systemic injection of MSCs also inhibits laser-induced retinal cell apoptosis, reduces the inflammatory response, improves the retinal tissue structure and limits the spread of damage. The therapeutic effect of MSCs administrated systemically may be attributed to the regulation of the intraocular microenvironment. Intravenous injection of MSCs is more practical than local injection in the clinic, because it is a simple procedure with no damage to the eye and allows flexible control of the cell dose. Hence, intravenous transplantation of MSCs is a promising therapeutic approach for the treatment of retinal trauma induced by laser exposure or other causal factors. An electroretinogram may be used to further determine whether MSCs improve or restore visual function, and the mechanisms of such effects need to be explored in detail.

## Abbreviations

AMD, age-related macular degeneration; MSCs, mesenchymal stem cells; GFP, green fluorescent protein; GFAP, glial fibrillary acidic protein; MMP-2, matrix metalloproteinase-2; ONL, outer nuclear layer; GCL, ganglion cell layer; INL, inner nuclear layer; RPE, retinal pigment epithelium; TUNEL, terminal deoxynucleotidyl transferase-mediated dUTP-biotin nick-end labeling; RT-PCR, reverse transcriptase-polymerase chain reaction; ECM, extracellular matrix.
